# Unraveling the Association Between Novel Lipid Biomarkers and Metabolic Syndrome: A Cross-Sectional Study

**DOI:** 10.7759/cureus.97623

**Published:** 2025-11-24

**Authors:** Bijoya Chatterjee, Hardik N Mahant, Prasanta Chatterjee Biswas, Jay Nagda, Nikunj Modi

**Affiliations:** 1 Biochemistry, Shri M. P. Shah Government Medical College, Jamnagar, IND; 2 Biochemistry, Medical College Baroda, Vadodara, IND; 3 Centre for Distance and Online Education, Parul University, Vadodara, IND; 4 Community Medicine, Shri M. P. Shah Government Medical College, Jamnagar, IND

**Keywords:** apolipoprotein a-i, apolipoprotein b, cardiovascular risk, comprehensive lipid tetrad index, lipoprotein(a), metabolic syndrome

## Abstract

Background

Metabolic syndrome (MetS) is a major public-health concern that substantially increases the risk of cardiovascular disease and type 2 diabetes. Traditional lipid profiles may not fully capture the atherogenic burden in MetS, prompting investigation of novel lipid biomarkers, such as lipoprotein(a) (Lp(a)), apolipoproteins, and the Comprehensive Lipid Tetrad Index (CLTI). This study evaluated the association of these biomarkers with MetS in an Indian outpatient population.

Methods

In a cross-sectional study of 707 adults aged 25-75 years at a tertiary care hospital in Jamnagar, Gujarat, MetS was diagnosed using NCEP-ATP III criteria. Serum Lp(a), apolipoprotein A-I (Apo A-I), apolipoprotein B (Apo B), and CLTI were measured using standard methods. Associations were tested using Chi-square analyses and logistic regression, and diagnostic performance was assessed by receiver operating characteristic (ROC) curve analysis.

Results

MetS was present in 397/707 (56.15%) participants, occurring in 197/332 (59.34%) of females and 200/375 (53.33%) of males. Elevated Lp(a), elevated Apo B, elevated CLTI, and reduced Apo A-I were all significantly associated with MetS (p < 0.001 for each). ROC analysis demonstrated the highest diagnostic accuracy for CLTI (area under the curve, or AUC = 0.835, 95% CI 0.806-0.862), followed by Lp(a) (AUC = 0.760, 95% CI 0.730-0.794), Apo B (AUC = 0.700, 95% CI 0.665-0.734), and Apo A-I (AUC = 0.620, 95% CI 0.584-0.657). Multivariable logistic regression identified elevated blood pressure, low high-density lipoprotein cholesterol, and elevated triglycerides as significant predictors of abnormal biomarker levels.

Conclusion

In this Indian outpatient cohort, CLTI and Lp(a) showed strong predictive value for MetS, and outperformed Apo A-I and Apo B when used alone. Incorporating CLTI and Lp(a) into clinical assessment may improve early detection and risk stratification in individuals at risk of MetS.

## Introduction

Metabolic syndrome (MetS) is a multifactorial disorder characterized by a cluster of interrelated risk factors - central obesity, dyslipidemia, hypertension, and insulin resistance - that together increase the risk of cardiovascular disease (CVD) and type 2 diabetes mellitus (T2DM) [1]. The global rise in MetS parallels increasing obesity and sedentary lifestyles. In India, rapid urbanization and lifestyle transitions have contributed to a marked increase in MetS prevalence. For example, analyses report an overall prevalence of approximately 30% in adults, increasing from about 13% in the 18-29 years cohort to roughly 50% in the 50-59 years cohort, depending on the population studied [2,3]. This upward trend has important implications for morbidity and mortality from CVD and T2DM.

Insulin resistance - a reduced cellular response to insulin - is central to MetS pathophysiology and leads to hyperglycemia, increased visceral adiposity, dysregulated lipid metabolism, and elevated blood pressure [4]. Traditional lipid measurements (total cholesterol (TC), low-density lipoprotein cholesterol (LDL-C), high-density lipoprotein cholesterol (HDL-C), triglycerides (TG)) are useful but may fail to capture important atherogenic features common in MetS, such as elevated TG and small, dense LDL particles, which can exist despite “normal” LDL-C levels [5].

To improve risk stratification, attention has shifted to novel lipid biomarkers, including apolipoprotein B (Apo B), apolipoprotein A-I (Apo A-I), and lipoprotein(a) (Lp(a)). Apo B reflects the number of atherogenic lipoprotein particles because each such particle contains a single Apo B molecule, and elevated Apo B strongly predicts CVD risk beyond LDL-C alone [6,7]. Apo A-I - the major protein of HDL particles - is central to reverse cholesterol transport, and lower Apo A-I levels have been linked to increased atherosclerotic risk [8]. Lp(a), an LDL-like particle linked to apolipoprotein(a), is largely genetically determined and is an independent risk factor for CVD; some studies also associate elevated Lp(a) with components of MetS [9].

Composite indices, such as the Comprehensive Lipid Tetrad Index (CLTI), which integrates traditional and novel lipid parameters, may offer superior risk discrimination in MetS. Indeed, studies show that relying solely on LDL-C can underestimate risk; for example, Paredes et al. found that a substantial proportion of MetS patients with optimal LDL-C had elevated Apo B, non-HDL-C, or oxidized LDL (OxLDL). Apo B and non-HDL-C correlated well with OxLDL, reinforcing their clinical value. Emerging markers (OxLDL, Apo B/Apo A-I ratio, and Lp(a)) also associate with inflammation and endothelial dysfunction, suggesting possible roles as therapeutic targets [10,11].

We, therefore, evaluated the association and predictive performance of Lp(a), Apo A-I, Apo B, and CLTI for MetS in an Indian tertiary-care outpatient population.

## Materials and methods

Study design and setting

This cross-sectional study was conducted over 3.5 years (December 2011-January 2014) at Shri M. P. Shah Government Medical College, Jamnagar, India, and participants were recruited through outpatient clinics at Sri Guru Gobind Singh Government Hospital. Ethical clearance was obtained from the Institutional Ethical Committee (approval no. MCLJ/IEC/13/10/2012) prior to recruitment.

Study population and sampling

Of 707 participants screened and enrolled using stratified random sampling (to balance age and sex strata), 397 fulfilled criteria for MetS. Participants were recruited from outpatient clinics at Sri Guru Gobind Singh Government Hospital between December 2011 and January 2014. We used stratified random sampling to ensure representation across sex (male/female) and age strata (25-45, 46-65, and >65). The sampling frame comprised outpatient registrants drawn daily; within each stratum, we used simple random sampling (random number generator) of clinic attendees who met eligibility criteria. The sampling approach ensured representation across age groups and both sexes.

Inclusion and exclusion criteria

The inclusion criteria are males and females aged 25-75 years; MetS diagnosis according to NCEP-ATP III criteria (≥3 of the following: elevated waist circumference (WC) - ≥90 cm for men and ≥80 cm for women; BP ≥130/85 mmHg or antihypertensive use; fasting blood glucose ≥100 mg/dL or antidiabetic therapy; TG ≥150 mg/dL; HDL-C <40 mg/dL in men and <50 mg/dL in women).

The exclusion criteria are current lipid-lowering therapy, pregnancy or lactation, and chronic renal or hepatic disease.

Data collection and clinical assessment

A structured questionnaire captured demographics, medical history, smoking, alcohol use, physical activity, and medication (Appendix). Clinical measures included height, weight, waist and hip circumferences, and blood pressure (average of two readings after five minutes’ rest). BMI was calculated as kg/m².

Biochemical assays

After an overnight fast (≥12 hours), venous blood was collected by trained phlebotomists. Serum Lp(a) was measured using a latex-enhanced immunoturbidimetric assay (Manufacturer: Roche Diagnostics, Basel, Switzerland; Tina-quant Lipoprotein(a) Gen.2) on an automated analyzer (Cobas c501, Roche Diagnostics). The assay lower limit of detection and inter-assay and intra-assay coefficients of variation (CV) were: LOD = 3 mg/dL, intra-assay CV = 1.8%, inter-assay CV = 2.4%. Apo A-I and Apo B concentrations were measured using immunoturbidimetric kits (Manufacturer: Roche Diagnostics, Basel, Switzerland; Tina-quant Apolipoprotein A-I ver.2 and Tina-quant Apolipoprotein B ver.2), with intra- and inter-assay CVs of 1.5% and 2.2% for Apo A-I, and 1.7% and 2.5% for Apo B, respectively. Lipid profile (TC, TG, HDL-C, and LDL-C) was measured using enzymatic colorimetric methods on the same platform (Cobas c501). All assays were performed according to manufacturer protocols, and laboratory personnel were blinded to the participants' clinical status. Samples were stored at -80°C if not analyzed the same day. Units are reported in mg/dL throughout. Where literature uses different units (e.g., nmol/L for Lp(a)), values are presented in mg/dL.

Biomarker cutpoints and rationale

We used previously published, reference-based cutoffs for categorizing biomarkers when available. Specifically: Apo A-I ≥120 mg/dL (normal) and <120 mg/dL (low); Apo B <90 mg/dL (normal) and ≥90 mg/dL (elevated); and Lp(a) ≥30 mg/dL (elevated) - cutoffs cited from established cardiovascular risk-assessment guidelines [12].

Where receiver operating characteristic (ROC) analysis identified an empirical optimal threshold (Youden index), we report that threshold and explicitly state that it was derived from our data. Differences between predefined clinical cutoffs and ROC-derived thresholds are discussed in the Results and Discussion; rounding and threshold conventions are explained (e.g., ROC optimal = 19.3 mg/dL, but we report ≥20 mg/dL for clinical interpretability).

Statistical analysis

Analyses used IBM SPSS Statistics for Windows, Version 26 (Released 2018; IBM Corp., Armonk, NY, USA). Descriptive data are reported as mean ± SD for continuous variables (or median with interquartile range where distribution was skewed) and n (%) for categorical variables. Between-group comparisons used analysis of variance (ANOVA) for normally distributed continuous variables or Kruskal-Wallis/Mann-Whitney tests for nonparametric continuous variables; categorical comparisons used Chi-square tests. Pearson correlation coefficients evaluated associations among continuous markers. Univariate and multivariate logistic regression models assessed independent predictors of elevated biomarkers and of MetS; stepwise selection adjusted for potential confounders. ROC curve analysis assessed the diagnostic performance of Apo B, Apo A-I, Lp(a), and CLTI; areas under the curve (AUC) with 95% CIs are reported. A two-tailed p < 0.05 was considered statistically significant. 

Note on thresholds and cutpoint reporting

For categorical comparisons, we used pre-specified, literature- or laboratory-based cutoffs (e.g., Apo A-I ≥121 mg/dL; Apo B ≥90 mg/dL; Lp(a) ≥20 mg/dL; CLTI ≥20,000), as described in the Methods. In addition, diagnostic performance was evaluated using ROC analysis and the Youden index to identify empiric optimal cutpoints from our sample. Where the ROC-derived cutpoint differed from the pre-specified clinical cutoff (for example, a ROC optimum of 19.x mg/dL for Lp(a) vs. a clinical cutoff of 20 mg/dL), both values are reported and discussed; rounding decisions and the rationale for choosing clinically interpretable thresholds are described in the Results and Discussion.

## Results

Table 1 presents the demographic, dietary, and clinical characteristics of 707 participants, categorized by sex. The majority were aged 46-65 years (57.28%, n = 405). A predominantly vegetarian diet was observed (78.64%, n = 556). MetS was present in 56.15% of individuals (n = 397), with a higher prevalence in females (59.33%, n = 197). Obesity (BMI >25) was found in 36.77% (n = 260), and high WC in 72.84% (n = 515). Elevated blood pressure was present in 60.25% (n = 426), and low HDL-C in 56.15% (n = 397). Coronary artery disease (CAD) with MetS (MetS + CAD) was diagnosed in 25.17% (n = 178). The data highlight key metabolic and cardiovascular risk factors in the study population.

**Table 1:** Demographic characteristics of the study population WC: Waist Circumference; BP: Blood Pressure; FBG: Fasting Blood Glucose; TG: Triglycerides; HDL-C: High-Density Lipoprotein Cholesterol; BMI: Body Mass Index; MetS: Metabolic Syndrome; CAD: Coronary Artery Disease

Characteristics	Females n (%), (n = 332)	Males n (%), (n = 375)	Total n (%), (n = 707)
Age Group			
25-45 years	114 (34.33%)	116 (30.93%)	230 (32.54%)
46-65 years	187 (56.33%)	218 (58.13%)	405 (57.28%)
>65 years	31 (9.34%)	41 (10.94%)	72 (10.18%)
Dietary Habit			
Vegetarian Diet	270 (81.20%)	286 (76.26%)	556 (78.64%)
Mixed Diet	62 (18.67%)	89 (23.73%)	151 (21.35%)
MetS			
Absent	135 (40.66%)	175 (46.66%)	310 (43.84%)
Present	197 (59.33%)	200 (53.33%)	397 (56.15%)
BMI (kg/m²)			
18.5-22.9	131 (40.66%)	161 (42.93%)	292 (41.30%)
23-24.9	70 (21.08%)	85 (22.66%)	155 (21.92%)
>25	131 (39.45%)	129 (34.40%)	260 (36.77%)
High WC (cm)			
Absent	57 (17.16%)	135 (37.06%)	192 (27.15%)
Present	275 (82.83%)	240 (64.00%)	515 (72.84%)
Elevated BP (mmHg)			
Absent	142 (42.77%)	139 (41.86%)	281 (39.74%)
Present	190 (57.22%)	236 (62.93%)	426 (60.25%)
Elevated FBG (mg/dL)			
Absent	212 (63.85%)	236 (62.93%)	551 (77.93%)
Present	120 (36.14%)	139 (37.06%)	156 (22.06%)
Elevated TG (mg/dL)			
Absent	214 (64.45%)	213 (56.80%)	427 (60.39%)
Present	118 (35.54%)	162 (43.20%)	280 (39.60%)
Low HDL-C (mg/dL)			
Absent	135 (40.66%)	175 (46.66%)	310 (43.84%)
Present	197 (59.33%)	200 (53.33%)	397 (56.15%)
Diagnosis			
MetS - CAD	20 (5.84%)	17 (4.50%)	37 (5.23%)
MetS + CAD	74 (22.30%)	104 (27.73%)	178 (25.17%)

A total of 397 participants with MetS were included in the study (197 females and 200 males). The distribution of MetS components among participants is detailed in Table 2. The majority were in the 46-65 years age group (61.2%, n = 243), followed by 25-45 years (28.5%, n = 113) and >65 years (10.3%, n = 41). High WC was observed in 77.8% (n = 309) of participants - higher in females (87.8%, n = 173) than in males (68.0%, n = 136). Elevated blood pressure was detected in 86.6% (n = 344) of those with MetS; hypertriglyceridemia (TG >150 mg/dL) was present in 62.7% (n = 249). Low HDL-C was present in 75.1% of participants with MetS (n = 298), more frequently in females (86.8%, n = 171) than in males (63.5%, n = 127) (Table 2).

**Table 2:** Gender wise prevalence (%) of various components in participants with MetS WC: Waist Circumference; BP: Blood Pressure; FBG: Fasting Blood Glucose; TG: Triglycerides; HDL-C: High-Density Lipoprotein Cholesterol; BMI: Body Mass Index; MetS: Metabolic Syndrome

MetS Components	Females n (%), (n = 197)	Males n (%), (n = 200)	Total n (%), (n = 397)
Age Group			
25-45 years	61 (31.00%)	52 (26.00%)	113 (28.50%)
46-65 years	117 (59.40%)	126 (63.00%)	243 (61.20%)
>65 years	19 (9.60%)	22 (11.00%)	41 (10.30%)
BMI (kg/m²)			
18.5-22.9	65 (33.00%)	67 (33.50%)	132 (33.20%)
23-24.9	37 (18.80%)	38 (19.00%)	75 (18.90%)
>25	95 (48.22%)	95 (47.50%)	190 (47.90%)
High WC (cm)			
Absent	24 (12.20%)	64 (32.00%)	88 (22.20%)
Present	173 (87.80%)	136 (68.00%)	309 (77.80%)
High BP (mmHg)			
Absent	32 (16.20%)	21 (10.50%)	53 (13.40%)
Present	165 (83.80%)	179 (89.50%)	344 (86.60%)
High FBG (mg/dL)			
Absent	84 (42.60%)	89 (44.50%)	173 (43.60%)
Present	113 (57.40%)	111 (55.50%)	224 (56.40%)
TG >150 mg/dL			
Absent	84 (42.60%)	64 (32.00%)	148 (37.30%)
Present	113 (57.40%)	136 (68.00%)	249 (62.70%)
Low HDL-C (mg/dL)			
Absent	26 (13.20%)	73 (36.50%)	99 (24.90%)
Present	171 (86.80%)	127 (63.50%)	298 (75.10%)

Table 3 shows gender-wise clinical comparisons among participants with MetS. Mean BMI was similar between females (25.09 ± 4.11 kg/m²) and males (25.25 ± 4.45 kg/m²; p = 0.97). Males had significantly higher mean WC (96.03 ± 9.14 cm) vs. females (92.37 ± 10.15 cm; p < 0.001). The waist-to-hip (WH) ratio was significantly higher in males (0.99 ± 0.07) than in females (0.91 ± 0.07; p < 0.001).

**Table 3:** Gender wise comparisons of clinical characteristics in MetS WC: Waist Circumference; WH Ratio: Waist-to-Hip Ratio; WHt Ratio: Waist-to-Height Ratio; SBP: Systolic Blood Pressure; DBP: Diastolic Blood Pressure; BMI: Body Mass Index; MetS: Metabolic Syndrome Continuous variables are presented as mean ± SD. p-values were obtained using Student’s t-test for between-group comparisons (female vs male).

Clinical Characteristics	Female (n = 197)	Male (n = 200)	p-value
Age (years)	52.66 ± 9.74	52.48 ± 10.64	0.82
BMI (kg/m^2^)	25.09 ± 4.11	25.25 ± 4.45	0.97
WC (cm)	92.37 ± 10.15	96.03 ± 9.14	<0.001
WH Ratio	0.91 ± 0.07	0.99 ± 0.07	<0.001
WHt Ratio	0.58 ± 0.06	0.57 ± 0.06	0.05
SBP (mmHg)	155.01 ± 22.28	158.29 ± 21.376	0.28
DBP (mmHg)	92.87 ± 9.84	94.78 ± 9.30	0.10

The distribution of novel lipid markers is summarized in Table 4. The median Lp(a) levels were comparable between females (22 mg/dL) and males (24.45 mg/dL; p = 0.34). Apo A-I levels were significantly lower in males (103.81 ± 21.21 mg/dL) than in females (109.00 ± 22.39 mg/dL; p = 0.029). No significant differences were observed in Apo B and CLTI levels.

**Table 4:** Novel lipid markers in participants MetS Lp(a) and CLTI are reported as median (IQR); Apo A-I and Apo B are reported as mean ± SD. p-values: Mann-Whitney U test for Lp(a) and CLTI; Student’s t-test for Apo A-I and Apo B. Significance was set at p < 0.05. Lp(a): Lipoprotein(a); Apo A-I: Apolipoprotein A-I; Apo B: Apolipoprotein B; CLTI: Comprehensive Lipid Tetrad Index; MetS: Metabolic Syndrome; IQR: Interquartile Range

Novel Lipid Markers	Female (n = 197)	Male (n = 200)	p-value
Lp(a) (mg/dL)	22 (36.25, 12.00)	24.45 (37.93, 12.55)	0.34
Apo A-1 (mg/dL)	109.00 ± 22.39	103.81 ± 21.21	0.029
Apo B (mg/dL)	107.59 ± 17.79	105.13 ± 20.04	0.203
CLTI (mg/dL)	18324.52 (30897.065, 8188.805)	20218.99 (32090.92, 9305.98)	0.262

Cross-tabulations show a significant association between elevated Lp(a) and MetS (Pearson χ² = 96.693, p < 0.001) (Tables 5-8). CLTI was strongly associated with MetS (Pearson χ² = 111.65, p < 0.001). Elevated Apo B showed a significant association with MetS (Pearson χ² = 68.869, p < 0.001).

**Table 5:** Lp(a) category vs MetS status (n = 707) Data are presented as n (%). Pearson Chi-square test was used. Pearson χ² = 96.693, df = 1, p < 0.001. Lp(a): Lipoprotein(a); MetS: Metabolic Syndrome

Lp(a) Category	No MetS (n = 310)	Yes MetS (n = 397)	Total (n = 707)
Normal (<20 mg/dL)	233 (75.16%)	151 (38.05%)	384 (54.31%)
Elevated (≥20 mg/dL)	77 (24.84%)	246 (61.95%)	323 (45.69%)

**Table 6:** Apo A-I category vs MetS status (n = 707) Data are presented as n (%). Categories: normal ≥121 mg/dL, low <121 mg/dL. Pearson Chi-square test was used. Pearson χ² = 35.841, df = 1, p < 0.001. Apo A-I: Apolipoprotein A-I; MetS: Metabolic Syndrome

Apo A-I Category	No MetS (n = 310)	Yes MetS (n = 397)	Total (n = 707)
Normal (≥121 mg/dL)	176 (56.77%)	309 (77.83%)	485 (68.59%)
Low (<121 mg/dL)	134 (43.23%)	88 (22.17%)	222 (31.41%)

**Table 7:** Apo B category vs MetS status (n = 707) Data are presented as n (%). Categories: normal <90 mg/dL, elevated ≥90 mg/dL. Pearson Chi-square test was used. Pearson χ² = 68.869, df = 1, p < 0.001. Apo B: Apolipoprotein B; MetS: Metabolic Syndrome

Apo B Category	No MetS (n = 310)	Yes MetS (n = 397)	Total (n = 707)
Normal (<90 mg/dL)	248 (80.00%)	197 (49.62%)	445 (62.94%)
Elevated (≥90 mg/dL)	62 (20.00%)	200 (50.38%)	262 (37.06%)

**Table 8:** CLTI category vs MetS status (n = 707) Data are presented as n (%). Categories: normal <20000, elevated ≥20000. Pearson Chi-square test was used. Pearson χ² = 111.65, df = 1, p < 0.001. CLTI: Comprehensive Lipid Tetrad Index; MetS: Metabolic Syndrome

CLTI Category	No MetS (n = 310)	Yes MetS (n = 397)	Total (n = 707)
Normal (<20000)	273 (88.06%)	200 (50.38%)	473 (66.90%)
Elevated (≥20000)	37 (11.94%)	197 (49.62%)	234 (33.10%)

For categorical analyses in Table 8, we applied a conventional CLTI cutoff of ≥20000 to define “elevated” values, consistent with local laboratory convention. However, ROC analysis produced an empiric optimal threshold in this study based on the Youden index; this ROC-derived cutpoint is data-driven and optimizes sensitivity and specificity in our sample. Both the categorical cutoff and the ROC-derived threshold (for diagnostic performance) are reported.

Logistic regression indicated that high BP (AOR = 0.122, 95% CI: 0.057-0.259, p < 0.001), low HDL-C (AOR = 2.878, 95% CI: 1.747-4.740, p < 0.001), and elevated TG (AOR = 1.859, 95% CI: 1.144-3.019, p = 0.012) were significant predictors of elevated Lp(a) (Tables 9-12). Low HDL-C (AOR = 13.37, 95% CI: 3.528-50.626, p < 0.001) and elevated TG (AOR = 5.02, 95% CI: 2.095-12.005, p < 0.001) predicted low Apo A-I. For Apo B, high BP (AOR ≈ 13.15, p < 0.001) and elevated TG (AOR = 2.101, p < 0.001) were significant predictors.

**Table 9:** Logistic regression model for evaluation of risk factors for high serum Lp(a) concentration Data are expressed as COR/AOR (95% CI) with corresponding p-values. Model I = Crude Odds Ratio (COR); Models II-IV = Adjusted Odds Ratios (AOR) from stepwise multivariable logistic regression models. Reference categories: Age 25-45 years, WC Absent, BP Normal, FBG Normal, HDL-C Normal, TG Normal. Statistical test: Binary logistic regression (*p-value < 0.05, indicating statistical significance). AOR: Adjusted Odds Ratio; CI: Confidence Interval; WC: Waist Circumference; BP: Blood Pressure; FBG: Fasting Blood Glucose; HDL-C: High-Density Lipoprotein Cholesterol; TG: Triglycerides; Lp(a): Lipoprotein(a); CLTI: Comprehensive Lipid Tetrad Index; MetS: Metabolic Syndrome

MetS Component	Model ICOR (95% CI)	p-value	Model II AOR (95% CI)	p-value	Model III AOR (95% CI)	p-value	Model IV AOR (95% CI)	p-value
Age Group (years)								
25-45	1.00 (Ref)	-	1.00 (Ref)	-	1.00 (Ref)	-	1.00 (Ref)	-
46-65	2.119 (0.923-4.863)	0.077	0.845 (0.516-1.383)	0.503	0.876 (0.533-1.440)	0.602	0.893 (0.534-1.494)	0.667
≥66	1.787 (0.815-3.919)	0.147	0.473 (0.206-1.086)	0.077	0.471 (0.204-1.085)	0.077	0.521 (0.220-1.235)	0.139
High WC (cm)								
Present	1.356 (0.797-2.309)	0.262	1.386 (0.812-2.368)	0.232	1.628 (0.930-2.849)	0.088	1.746 (0.978-3.117)	0.060
Absent	1.00 (Ref)	-	1.00 (Ref)	-	1.00 (Ref)	-	1.00 (Ref)	-
High BP (mmHg)								
Present	0.095 (0.046-0.197)	<0.001*	0.095 (0.046-0.197)	<0.001*	0.098 (0.047-0.206)	<0.001*	0.122 (0.057-0.259)	<0.001*
Absent	1.00 (Ref)	-	1.00 (Ref)	-	1.00 (Ref)	-	1.00 (Ref)	-
Elevated FBG (mg/dL)								
Present	1.285 (0.822-2.009)	0.272	1.287 (0.823-2.014)	0.269	1.349 (0.858-2.122)	0.195	12.392 (5.425-28.307)	<0.001*
Absent	1.00 (Ref)	-	1.00 (Ref)	-	1.00 (Ref)	-	1.00 (Ref)	-
Low HDL-C (mg/dL)								
Present	2.789 (1.763-4.413)	<0.001*	2.903 (1.819-4.634)	<0.001*	2.791 (1.742-4.469)	<0.001*	2.878 (1.747-4.740)	<0.001*
Absent	1.00 (Ref)	-	1.00 (Ref)	-	1.00 (Ref)	-	1.00 (Ref)	-
Elevated TG (mg/dL)								
Present	1.589 (1.021-2.502)	0.040*	1.583 (1.009-2.483)	0.045*	1.578 (1.003-2.482)	0.048*	1.859 (1.144-3.019)	0.012*
Absent	1.00 (Ref)	-	1.00 (Ref)	-	1.00 (Ref)	-	1.00 (Ref)	-

**Table 10:** Logistic regression model for evaluation of risk factors for low serum Apo A-I concentration Data are expressed as COR/AOR (95% CI) with corresponding p-values. Model I = Crude Odds Ratio (COR); Models II-IV = Adjusted Odds Ratios (AOR) from multivariable logistic regression models. Reference categories: Age 25-45 years, WC Absent, BP Normal, FBG Normal, HDL-C Normal, TG Normal. Statistical test: Binary logistic regression (*p-value < 0.05, indicating statistical significance). AOR: Adjusted Odds Ratio; CI: Confidence Interval; WC: Waist Circumference; BP: Blood Pressure; FBG: Fasting Blood Glucose; HDL-C: High-Density Lipoprotein Cholesterol; TG: Triglycerides; Apo A-I: Apolipoprotein A-I; CLTI: Comprehensive Lipid Tetrad Index; MetS: Metabolic Syndrome

MetS Component	Model I COR (95% CI)	p-value	Model II AOR (95% CI)	p-value	Model III AOR (95% CI)	p-value	Model IV AOR (95% CI)	p-value
Age Group (years)								
25-45	1.00 (Ref)	-	1.00 (Ref)	-	1.00 (Ref)	-	1.00 (Ref)	-
46-65	2.110 (0.616-7.239)	0.240	2.109 (0.615-7.234)	0.235	2.360 (0.674-8.247)	0.180	3.596 (0.895-14.438)	0.071
≥66	1.230 (0.413-3.666)	0.710	1.230 (0.413-3.667)	0.710	1.440 (0.427-4.380)	0.523	2.140 (0.633-7.232)	0.221
High WC (cm)								
Present	0.940 (0.624-1.417)	0.769	0.974 (0.642-1.478)	0.902	1.170 (0.758-1.818)	0.473	1.380 (0.861-2.200)	0.182
Absent	1.00 (Ref)	-	1.00 (Ref)	-	1.00 (Ref)	-	1.00 (Ref)	-
High BP (mmHg)								
Present	65.400 (8.800-486.032)	<0.001*	66.315 (8.865-496.099)	<0.001*	61.220 (8.104-462.448)	<0.001*	37.380 (4.859-287.550)	0.001*
Absent	1.00 (Ref)	-	1.00 (Ref)	-	1.00 (Ref)	-	1.00 (Ref)	-
Elevated FBG (mg/dL)								
Present	2.593 (1.316-5.108)	0.006*	2.689 (1.350-5.356)	0.005*	2.610 (1.295-5.275)	0.070	2.260 (1.028-4.965)	0.043*
Absent	1.00 (Ref)	-	1.00 (Ref)	-	1.00 (Ref)	-	1.00 (Ref)	-
Low HDL-C (mg/dL)								
Present	9.512 (3.473-26.051)	<0.001*	12.604 (4.235-37.514)	<0.001*	12.860 (4.153-39.785)	<0.001*	13.370 (3.528-50.626)	<0.001*
Absent	1.00 (Ref)	-	1.00 (Ref)	-	1.00 (Ref)	-	1.00 (Ref)	-
Elevated TG (mg/dL)								
Present	7.926 (3.751-16.749)	<0.001*	8.931 (4.084-19.533)	<0.001*	8.320 (3.783-18.315)	<0.001*	5.020 (2.095-12.005)	<0.001*
Absent	1.00 (Ref)	-	1.00 (Ref)	-	1.00 (Ref)	-	1.00 (Ref)	-

**Table 11:** Logistic regression model for evaluation of risk factors for high serum Apo B concentration Data are expressed as COR/AOR (95% CI) with corresponding p-values. “†" indicates missing or not provided values. Model I = Crude Odds Ratio (COR); Models II-IV = Adjusted Odds Ratios (AOR) from multivariable logistic regression models. Reference categories: Age 25-45 years, WC Absent, BP Normal, FBG Normal, HDL-C Normal, TG Normal. Statistical test: Binary logistic regression (*p-value < 0.05, indicating statistical significance). AOR: Adjusted Odds Ratio; CI: Confidence Interval; WC: Waist Circumference; BP: Blood Pressure; FBG: Fasting Blood Glucose; HDL-C: High-Density Lipoprotein Cholesterol; TG: Triglycerides; Apo B: Apolipoprotein B; CLTI: Comprehensive Lipid Tetrad Index; MetS: Metabolic Syndrome

MetS Component	Model I COR (95% CI)	p-value	Model II AOR (95% CI)	p-value	Model III AOR (95% CI)	p-value	Model IV AOR (95% CI)	p-value
Age Group (years)								
25-45	1.00 (Ref)	-	1.00 (Ref)	-	1.00 (Ref)	-	1.00 (Ref)	-
46-65	2.282 (1.526-5.440)	0.001*	2.809 (1.485-5.314)	0.001*	2.776 (1.456-5.291)	0.002*	2.551 (1.283-5.071)	0.008*
≥66	2.327 (1.289-4.201)	0.005*	2.294 (1.269-4.146)	0.006*	2.353 (1.292-4.286)	0.005*	2.154 (1.137-4.081)	0.019*
High WC (cm)								
Present	1.135 (0.755-1.705)	0.544	1.243 (0.817-1.891)	0.311	1.502 (0.967-2.332)	0.070	1.730 (1.085-2.759)	0.021*
Absent	1.00 (Ref)	-	1.00 (Ref)	-	1.00 (Ref)	-	1.00 (Ref)	-
High BP (mmHg)								
Present	18.889 (10.112-35.283)	<0.001*	18.541 (9.861-34.862)	<0.001*	17.684 (9.334-33.502)	<0.001*	13.154 (2.53- not given†)	<0.001*
Absent	1.00 (Ref)	-	1.00 (Ref)	-	1.00 (Ref)	-	1.00 (Ref)	-
Elevated FBG (mg/dL)								
Present	1.919 (1.332-2.769)	<0.001*	1.936 (1.339-2.798)	<0.001*	2.101 (1.440-3.064)	<0.001*	1.988 (1.335-2.959)	0.001*
Absent	1.00 (Ref)	-	1.00 (Ref)	-	1.00 (Ref)	-	1.00 (Ref)	-
Low HDL-C (mg/dL)								
Present	3.355 (1.962-5.735)	<0.001*	3.501 (2.033-6.030)	<0.001*	3.354 (1.940-5.798)	<0.001*	3.133 (1.759-5.578)	<0.001*
Absent	1.00 (Ref)	-	1.00 (Ref)	-	1.00 (Ref)	-	1.00 (Ref)	-
Elevated TG (mg/dL)								
Present	3.134 (2.166-4.534)	<0.001*	3.046 (2.100-4.420)	<0.001*	2.930 (2.012-4.268)	<0.001*	2.101 (1.404-3.143)	<0.001*
Absent	1.00 (Ref)	-	1.00 (Ref)	-	1.00 (Ref)	-	1.00 (Ref)	-

**Table 12:** Logistic regression analysis: association of MetS components with high CLTI Data are presented as COR/AOR (95% CI) and p-value. Model I = Crude Odds Ratio (COR); Models II-IV = Adjusted Odds Ratios (AOR) from stepwise multivariable logistic regression models. Reference categories: Age 25-45 years; Absent for binary MS components. *p-value < 0.05, indicating statistical significance. AOR: Adjusted Odds Ratio; CI: Confidence Interval; WC: Waist Circumference; BP: Blood Pressure; FBG: Fasting Blood Glucose; HDL-C: High-Density Lipoprotein Cholesterol; TG: Triglycerides; CLTI: Comprehensive Lipid Tetrad Index; MetS: Metabolic Syndrome

MetS Component	Model I - COR (95% CI)	p-value	Model II - AOR (95% CI)	p-value	Model III - AOR (95% CI)	p-value	Model IV - AOR (95% CI)	p-value
Age group (ref: 25-45)								
46-65	2.475 (0.887-6.907)	0.083	2.531 (0.904-7.087)	0.077	2.489 (0.887-6.982)	0.083	2.286 (0.807-6.475)	0.120
≥66	1.362 (0.514-3.609)	0.535	1.399 (0.526-3.720)	0.501	1.418 (0.532-3.781)	0.485	1.291 (0.479-3.481)	0.610
High WC (ref: Absent)								
Present	2.218 (1.046-4.702)	0.038*	2.417 (1.123-5.204)	0.024*	2.627 (1.197-5.764)	0.016*	2.635 (1.192-5.821)	0.020*
High BP (ref: Absent)								
Present	7.619 (3.018-19.232)	<0.001*	7.533 (2.976-19.071)	<0.001*	7.477 (2.923-19.127)	<0.001*	7.006 (2.719-18.050)	<0.001*
Elevated FBG (ref: Absent)								
Present	0.996 (0.573-1.731)	0.988	0.977 (0.599-1.708)	0.936	1.006 (0.573-1.766)	0.984	1.013 (0.574-1.788)	0.970
Low HDL-C (ref: Absent)								
Present	1.872 (1.064-3.294)	0.030*	2.019 (1.133-3.596)	0.017*	1.978 (1.104-3.544)	0.022*	2.378 (1.288-4.392)	0.010*
Elevated TG (ref: Absent)								
Present	1.098 (0.600-2.010)	0.762	1.122 (0.611-2.061)	0.710	1.104 (0.600-2.032)	0.751	1.113 (0.601-2.058)	0.730

Table 13 presents the ROC curve analysis for novel lipid markers. The AUC was highest for CLTI (AUC = 0.835, 95% CI: 0.806-0.862), followed by Lp(a) (AUC = 0.76, 95% CI: 0.730-0.794), Apo B (AUC = 0.70, 95% CI: 0.665-0.734), and Apo A-I (AUC = 0.62, 95% CI: 0.584-0.657). 

**Table 13:** ROC curve analysis of novel lipid markers in MetS population AUC: Area Under the Curve; SE: Standard Error; CI: Confidence Interval; Lp(a): Lipoprotein(a); Apo A-I: Apolipoprotein A-I; Apo B: Apolipoprotein B; CLTI: Comprehensive Lipid Tetrad Index; MetS: Metabolic Syndrome

Novel Lipid Markers	AUC	SE	95% CI	Specificity	Sensitivity	Criterion
Lp(a) (mg/dL)	0.76	0.0196	0.730, 0.794	78.53	84.57	≤19
Apo A-I (mg/dL)	0.62	0.0234	0.584, 0.657	47.88	59.04	≥117
Apo B (mg/dL)	0.7	0.0221	0.665, 0.734	75.29	74.47	≤108
CLTI (mg/dL)	0.835	0.0154	0.806, 0.862	90.96	80.46	>11061.36

**Figure 1:**
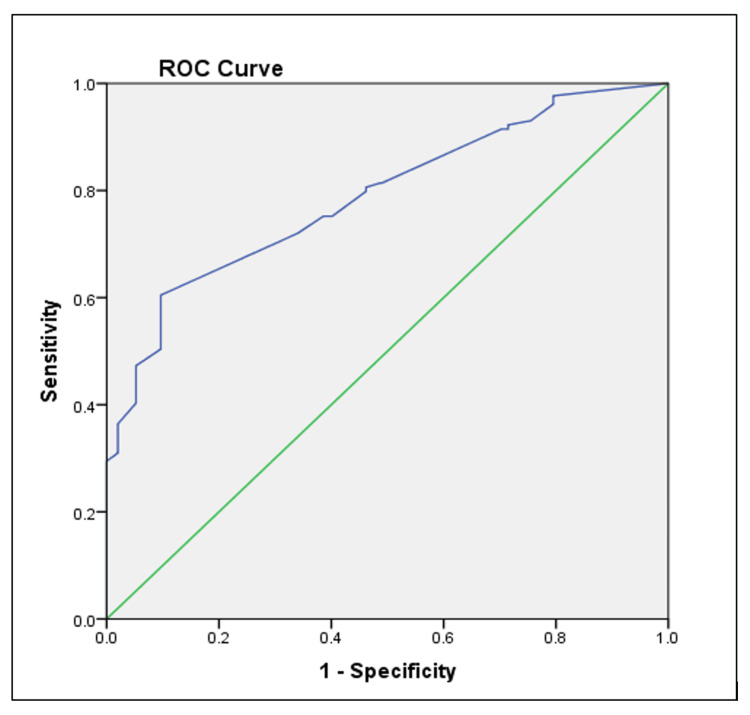
ROC curve of Lp(a) ratios in MetS This ROC curve illustrates the diagnostic performance of Lp(a) ratios in identifying subjects with metabolic syndrome. The curve demonstrates the trade-off between sensitivity and specificity at various thresholds, indicating moderate discriminatory power. Lp(a): Lipoprotein(a); MetS: Metabolic Syndrome; ROC: Receiver Operating Characteristic

Figure 1 illustrates the ROC curve for Lp(a), showing its predictive capacity for MetS.

**Figure 2:**
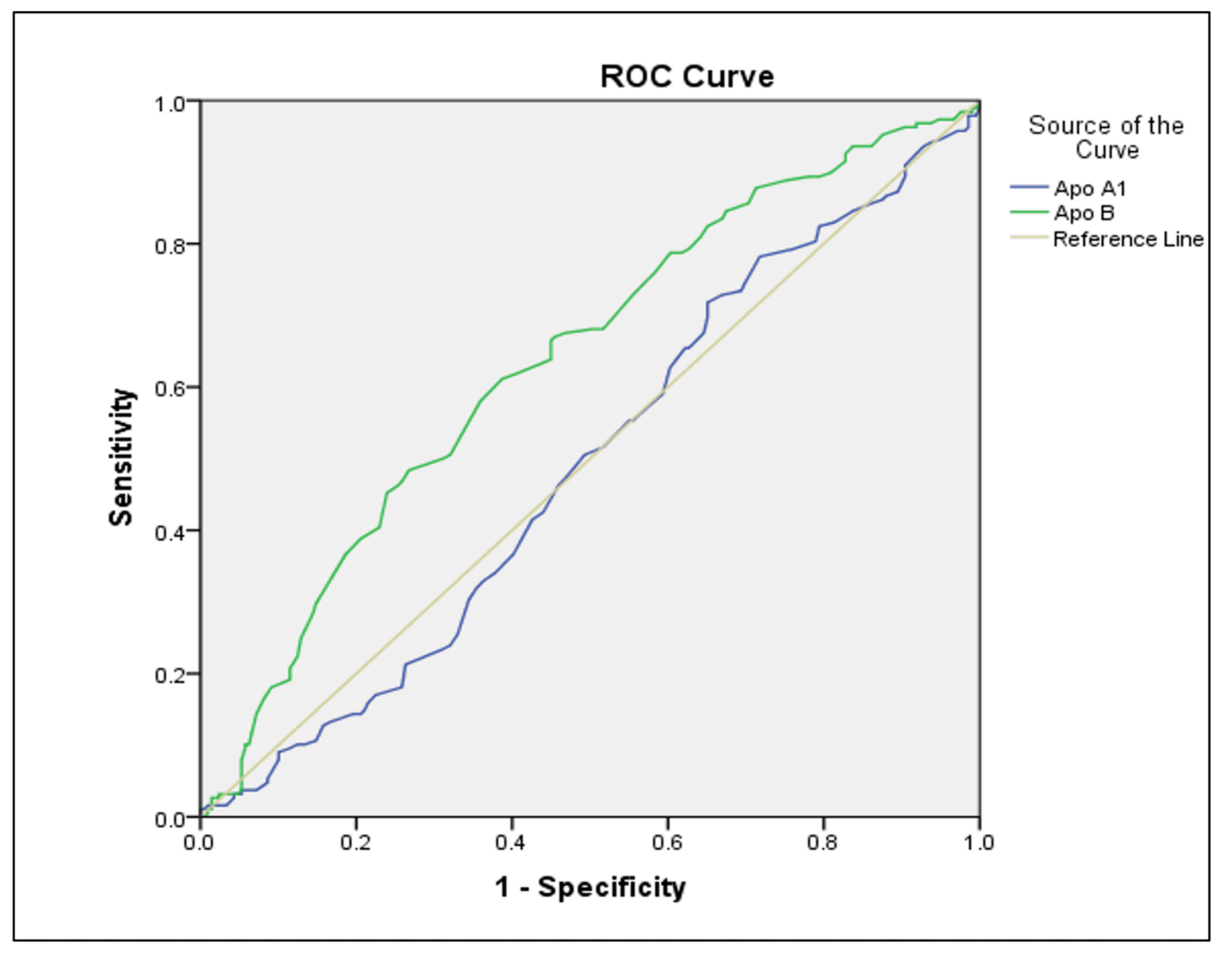
ROC curve of Apo A-I and Apo B in MetS This figure compares the ROC curves of Apo A-I and Apo B in detecting metabolic syndrome. Apo B shows relatively higher sensitivity and specificity than Apo A-I, suggesting better diagnostic performance. The diagonal reference line indicates a test with no discriminatory ability. Apo A-I: Apolipoprotein A-I; Apo B: Apolipoprotein B; ROC: Receiver Operating Characteristic; MetS: Metabolic Syndrome

Figure 2 demonstrates the ROC curves for Apo A-I and Apo B, depicting their respective diagnostic accuracies.

**Figure 3:**
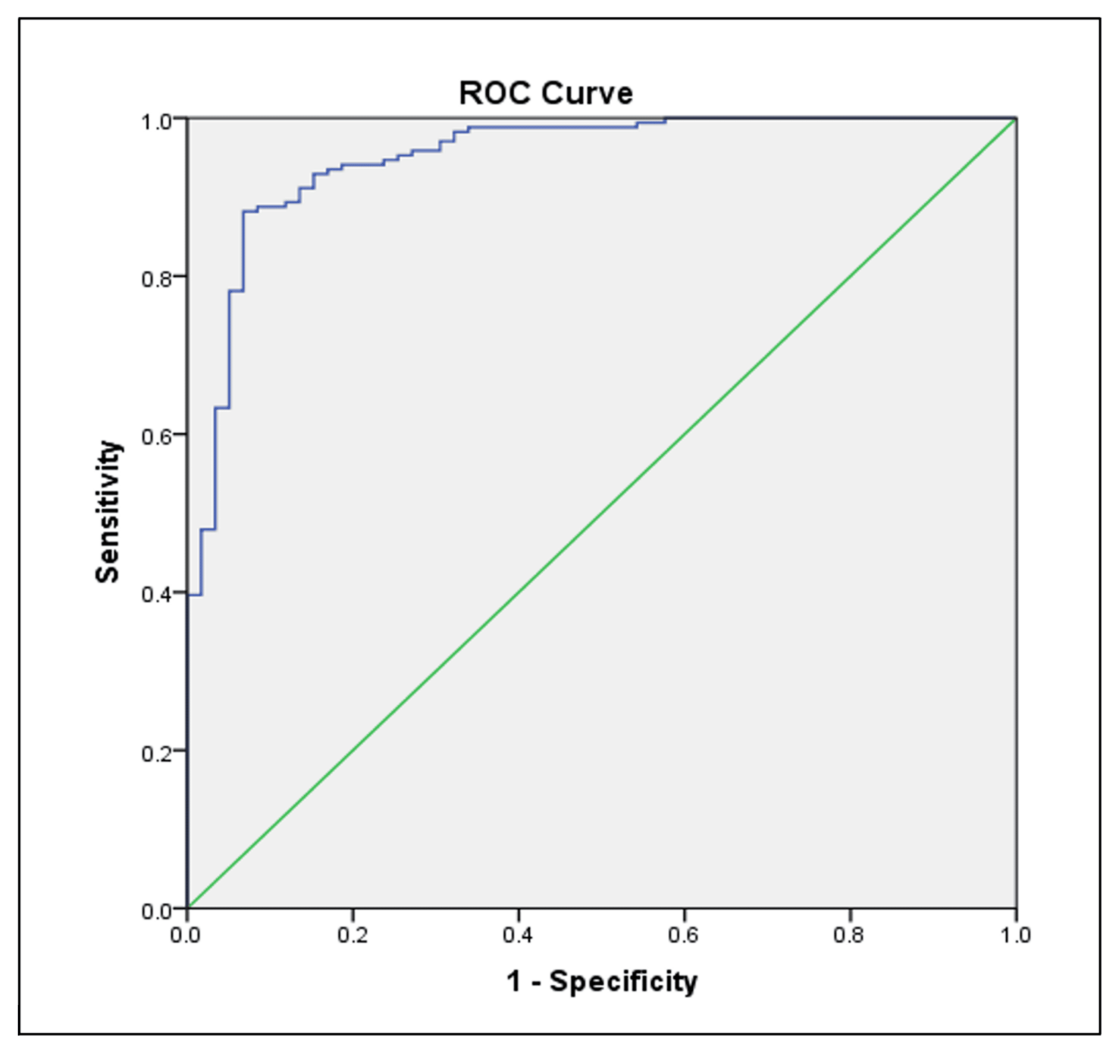
ROC curve of CLTI in MetS This ROC curve evaluates the diagnostic accuracy of the CLTI for metabolic syndrome. The curve is positioned well above the reference line, suggesting excellent predictive performance with high sensitivity and specificity. ROC: Receiver Operating Characteristic; MetS: Metabolic Syndrome; CLTI: Comprehensive Lipid Tetrad Index

Figure 3 displays the ROC curve for the CLTI, which had the highest AUC among all markers evaluated.

These findings suggest that CLTI and Lp(a) are stronger predictors of MetS compared to Apo A-I and Apo B.

## Discussion

This cross-sectional study provides valuable insights into the role of novel lipid biomarkers in predicting MetS among an Indian population. The findings demonstrate significant associations between these biomarkers and MetS, highlighting their potential as predictive tools for early detection and risk stratification.

The prevalence of MetS in our study population (56.15%) aligns with previous reports from urban Indian settings, reflecting the growing burden of metabolic disorders in the country [13]. This high prevalence underscores the urgent need for effective screening and preventive strategies.

Among the novel lipid biomarkers examined, Lp(a) emerged as a strong predictor of MetS. The significant association between elevated Lp(a) levels and MetS (χ² = 96.693, p < 0.001) corroborates findings from previous studies, suggesting Lp(a) as an independent risk factor for CVD [14]. The logistic regression analysis revealed that hypertension, low HDL-C, and elevated TG were significant predictors of high Lp(a) levels, indicating a complex interplay between Lp(a) and other metabolic risk factors.

Apo B levels were also significantly associated with MetS (χ² = 68.869, p < 0.001), consistent with its role as a marker of atherogenic lipoproteins. The strong predictive value of Apo B for MetS, particularly its association with hypertension and hypertriglyceridemia, supports its potential use as a more accurate indicator of cardiovascular risk than traditional lipid measures [5].

Interestingly, our study found lower levels of Apo A-I in males compared to females with MetS. This gender difference in Apo A-I levels may reflect underlying variations in HDL metabolism and could contribute to the differential cardiovascular risk observed between men and women [10].

The CLTI demonstrated the highest predictive accuracy for MetS among all biomarkers studied (AUC = 0.835). This composite index, which incorporates both traditional and novel lipid parameters, offers a more comprehensive assessment of lipid-related cardiovascular risk. The superior performance of CLTI suggests that integrative approaches to lipid profiling may provide enhanced risk stratification for MetS and related complications [15].

The ROC curve analysis revealed that CLTI and Lp(a) had higher predictive values for MetS compared to Apo A-I and Apo B. This finding underscores the potential of these markers, particularly CLTI, as screening tools for MetS in clinical practice. The implementation of these biomarkers could enhance early detection and facilitate targeted interventions to prevent MetS-related complications.

Additionally, systemic oxidative stress has been increasingly recognized as a pivotal contributor to the pathogenesis of MetS. OxLDL, a marker of oxidative stress, has been shown to be elevated in individuals with MetS and is implicated in vascular dysfunction and atherosclerosis. Incorporating oxidative biomarkers, like OxLDL, as part of a biomarker panel may provide greater diagnostic value in detecting MetS-related complications [16].

Gender-specific differences observed in our study, such as the higher prevalence of central obesity and low HDL-C in females, highlight the need for gender-tailored approaches in MetS prevention and management. These differences may be attributed to hormonal factors, body fat distribution, and lifestyle variations between men and women [17].

The significant associations between novel lipid biomarkers and individual components of MetS (e.g., hypertension and dyslipidemia) provide insights into the complex pathophysiology of the syndrome. These relationships suggest that lipid abnormalities play a central role in the development of MetS and its associated cardiovascular risk [4].

Our findings have important clinical implications. In this cross-sectional outpatient cohort, CLTI and Lp(a) were more strongly associated with MetS than Apo A-I and Apo B when evaluated individually. These findings suggest that CLTI and Lp(a) warrant further investigation as potential adjuncts for risk stratification. However, the cross-sectional design precludes causal inference, and our results are associative. Prospective, multicenter studies are needed to validate these biomarkers, determine optimal and generalizable cutpoints, and assess whether their incorporation into clinical algorithms improves prediction, prevention, or management of MetS-related outcomes.

Limitations 

Several limitations should be noted. First, the cross-sectional design prevents assessment of temporality or causality between lipid biomarkers and MetS. Second, the study was conducted at a single tertiary-care center, and the data were collected between December 2011 and January 2014; temporal changes in population risk factors or assay platforms may limit generalizability to present-day or geographically different cohorts. Third, assay-specific factors (manufacturer, lot, and analytical sensitivity) can influence biomarker values. Finally, while we report both literature-based and ROC-derived cutpoints, these thresholds require prospective validation before clinical adoption.

## Conclusions

This study demonstrates the significant predictive value of novel lipid biomarkers, particularly CLTI and Lp(a), for MetS in an Indian population. These findings support the incorporation of these markers into clinical practice for enhanced risk assessment and early intervention strategies. Future research should focus on validating these biomarkers in diverse populations and evaluating their utility in guiding therapeutic decisions for MetS management.

## Appendices

Structured questionnaire

*Section A: Demographic Information* 

1. Participant ID: ________ 

2. Age (years): ________ 

3. Sex: 

o ☐ Male 

o ☐ Female 

4. Occupation: 

o ☐ Homemaker 

o ☐ Laborer 

o ☐ Service 

o ☐ Business 

o ☐ Professional 

o ☐ Other: ________ 

5. Education Level: 

o ☐ No formal education 

o ☐ Primary 

o ☐ Secondary 

o ☐ Graduate 

o ☐ Postgraduate 

Section B: Lifestyle and Behavioral Factors

6. Dietary Pattern: 

o ☐ Vegetarian 

o ☐ Mixed 

7. Smoking Status: 

o ☐ Never 

o ☐ Former 

o ☐ Current

8. Alcohol Consumption: 

o ☐ Never 

o ☐ Occasional 

o ☐ Regular 

9. Physical Activity: 

o ☐ Sedentary 

o ☐ Moderate 

o ☐ Vigorous 

Section C: Medical History 

10. Known Hypertension: 

o ☐ Yes ☐ No 

11. On Antihypertensive Medication: 

o ☐ Yes ☐ No 

12. Known Diabetes Mellitus: 

o ☐ Yes ☐ No 

13. On Antidiabetic Medication: 

o ☐ Yes ☐ No 

14. History of Dyslipidemia: 

o ☐ Yes ☐ No 

15. History of Cardiovascular Disease (e.g., CAD): 

o ☐ Yes ☐ No 

16. Family History of CVD/Diabetes: 

o ☐ Yes ☐ No 

17. Current Medications (select all that apply): 

o ☐ Antihypertensives 

o ☐ Antidiabetics 

o ☐ Antiplatelets 

o ☐ Lipid-lowering drugs (statins/fibrates)* 

o ☐ Others: ________ 

(*Participants on lipid-lowering therapy were excluded.)

Section D: Clinical Measurements (recorded after 5 minutes rest)

18. Height (cm): ________ 

19. Weight (kg): ________ 

20. BMI (kg/m²): ________ 

21. Waist Circumference (cm): ________ 

22. Hip Circumference (cm): ________ 

23. Waist-to-Hip Ratio: ________ 

24. Waist-to-Height Ratio: ________ 

25. Blood Pressure (mmHg): 

• Reading 1: SBP ____ / DBP ____ 

• Reading 2: SBP ____ / DBP ____ 

• Average BP: SBP ____ / DBP ____ 

Section E: Laboratory Parameters (fasting ≥12 hours)

26. Fasting Blood Glucose (mg/dL): ________ 

27. Lipid Profile: 

• Total Cholesterol (mg/dL): ________ 

• Triglycerides (mg/dL): ________ 

• HDL-C (mg/dL): ________ 

• LDL-C (mg/dL): ________ 

28. Novel Lipid Biomarkers: 

• Lipoprotein(a) - Lp(a) (mg/dL): ________ 

• Apolipoprotein A-I - Apo A-I (mg/dL): ________ 

• Apolipoprotein B - Apo B (mg/dL): ________ 

• Comprehensive Lipid Tetrad Index (CLTI): 

Formula: CLTI = TC × TG × Lp(a) / Apo A-I 

Value: ________

Section F: Metabolic Syndrome (NCEP-ATP III Criteria) Assessed by Investigator

29. Increased Waist Circumference 

• ☐ Present (≥90 cm in men; ≥80 cm in women) 

• ☐ Absent 

30. Elevated Blood Pressure 

• ☐ Present (≥130/85 mmHg or on treatment) 

• ☐ Absent 

31. Fasting Blood Glucose ≥100 mg/dL 

• ☐ Present 

• ☐ Absent 

32. Triglycerides ≥150 mg/dL 

• ☐ Present 

• ☐ Absent 

33. Low HDL-C 

• ☐ Present (<40 mg/dL in men; <50 mg/dL in women) • ☐ Absent 

34. Final Classification: 

• ☐ Metabolic Syndrome (≥3 criteria present) 

• ☐ Not Metabolic Syndrome
